# What is the optimal duration of immune checkpoint inhibitors in malignant tumors?

**DOI:** 10.3389/fimmu.2022.983581

**Published:** 2022-09-26

**Authors:** Jiaxin Yin, Yuxiao Song, Jiazhuo Tang, Bicheng Zhang

**Affiliations:** Cancer Center, Renmin Hospital of Wuhan University, Wuhan, China

**Keywords:** duration, immune checkpoint inhibitors, immunotherapy, malignant tumor, optimization

## Abstract

Immunotherapy, represented by immune checkpoint inhibitors (ICIs), has made a revolutionary difference in the treatment of malignant tumors, and considerably extended patients’ overall survival (OS). In the world medical profession, however, there still reaches no clear consensus on the optimal duration of ICIs therapy. As reported, immunotherapy response patterns, immune-related adverse events (irAEs) and tumor stages are all related to the diversity of ICIs duration in previous researches. Besides, there lacks clear clinical guidance on the intermittent or continuous use of ICIs. This review aims to discuss the optimal duration of ICIs, hoping to help guide clinical work based on the literature.

## Introduction

In recent years, immune checkpoint inhibitors (ICIs), represented by programmed cell death protein-1 (PD-1)/programmed cell death ligand 1 (PD-L1) and cytotoxic T lymphocyte-associated protein 4 (CTLA-4) monoclonal antibodies, have revolutionized the treatment of malignant tumors. Consequently, patients are accessible to more treatment options and acquire longer overall survival (OS). Despite the significant efficacy, ICIs simultaneously trigger off a growing number of issues, such as the management of immune-related adverse events (irAEs), the mechanism and the management strategies of immunotherapy resistance, valid predictive biomarkers of ICIs treatment, the optimization of ICIs-based combination therapies and using ICIs in special populations, all of which not only puzzle both oncologists and patients but remain further exploration. Moreover, there exists no clear consensus on the optimal duration of ICIs therapy (1–4), about which an up-to-date review of the current cognition is presented here.

## Response patterns determine the duration of ICIs

**Figure 1:**
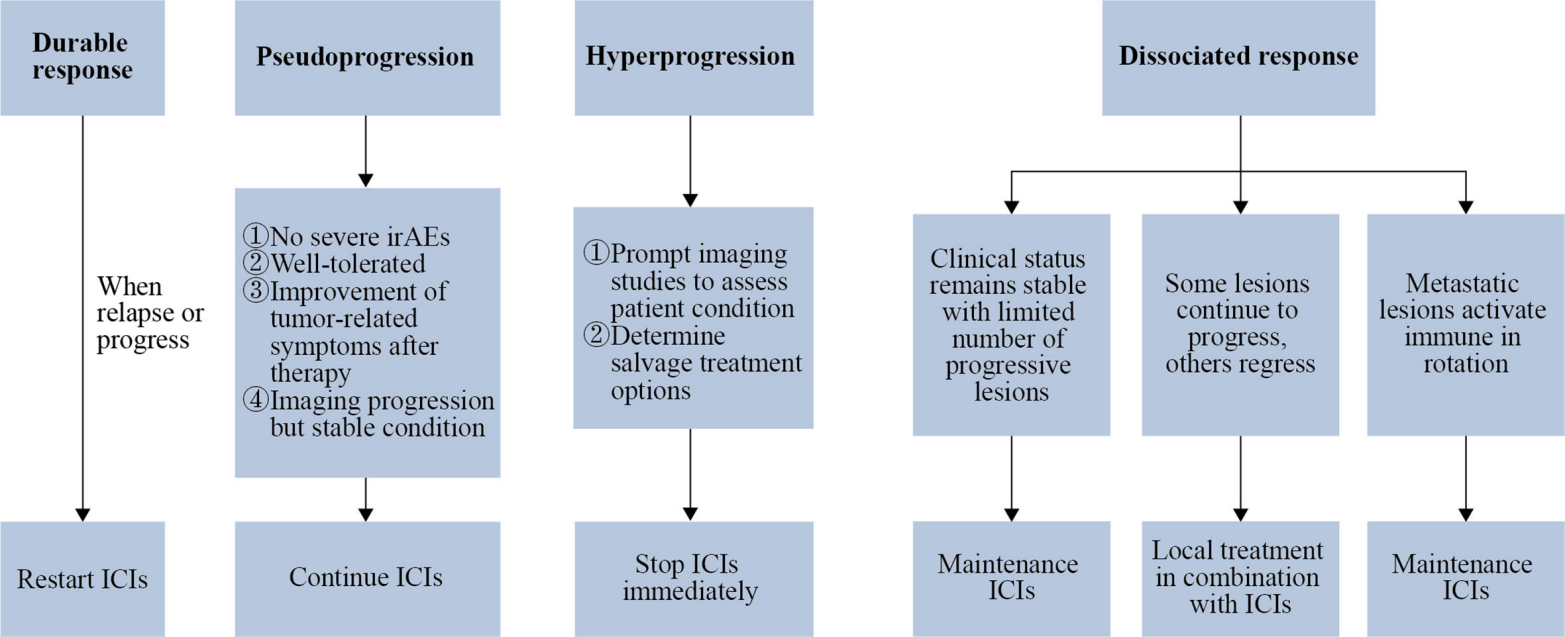
Response patterns determine the duration of ICIs. If there remains a durable response after ICIs cessation, restarting ICIs treatment may be considered in the situation of relapse or progression. Patients who match the exhibiting criteria can be considered for continuation of ICIs after being diagnosed with pseudoprogression. When hyperprogression is confirmed, ICIs treatment should be stopped as soon as possible, followed by radiologic examination to assess the patient’s condition and decide the treatment alternatives. As for patients with a dissociated response, when the clinical condition remains stable and the number of progressive lesions is limited, maintenance ICIs may be an option; when a minority of metastatic lesions continue to progress while the rest of the metastatic lesions are in remission, local treatment can be chosen in conjunction with ICIs treatment; when metastatic lesions activate immune in rotation, ICIs should be maintained without local treatment.

With the widespread clinical application of ICIs, it has been gradually found that only a fraction of patients treated with ICIs can achieve durable responses, which means significant and long-lasting curative effect. During ICIs treatment, a considerable percentage of patients exhibits alternative response patterns including pseudoprogression, hyperprogression and dissociated response (5, 6). The prognosis of patients significantly varies from different response patterns, and the duration of ICIs treatment needs to be adjusted accordingly (**Figure 1**).

### Durable response

Currently, the definition of durable response remains controversial. In a randomized phase III trial, the durable response was defined as follows: Progression-free survival (PFS) of a patient exceeded three times longer than the median PFS of the same group (7). Durable responses can persist for months or years in patients treated with ICIs, some of them even have improved responses to ICIs over time, usually bringing a longer OS (8).

According to published clinical consensus, patients with advanced malignant melanoma who have achieved both complete response (CR) and ICIs treatment for at least six months can consider ceasing ICIs. If the efficacy is assessed as partial response (PR) or stable disease (SD) after two years of ICIs treatment, cessation may be taken into account (1, 9, 10). PET-CT, liquid biopsy (e.g., ctDNA) or tissue biopsy are recommended options for determining efficacy evaluation during ICIs treatment. This consensus on ICIs duration is worth applying to the immunotherapy of other malignant tumors. However, a small number of trials have found that one year of nivolumab treatment for advanced non-small cell lung cancer (NSCLC) may be insufficient. More studies are exploring the feasibility of early discontinuing ICIs treatment, which aims to achieve less treatment-related toxicities and longer OS (11). Some researchers put forward that limited ICIs rather than continuous ICIs might be adequate to induce a durable response (12).

In addition, if there remains a durable response after ICIs cessation (including programmed cessation and cessation for reasons such as economic conditions), restarting ICIs treatment may be considered in the situation of relapse or progressive disease (PD). Clinical studies researching the efficacy of re-challenging ICIs after early discontinuation exist as well. In conclusion, the optimal duration of ICIs is still debatable for patients with a durable response and needs to be further explored with prospective studies.

### Pseudoprogression

The tumors can present a transient increase in volume or number of lesions (temporary progression) after ICIs treatment, followed by PR or SD, which is defined as pseudoprogression (13). Pseudoprogression was first identified in patients with metastatic melanoma treated with ipilimumab (14). Up to 10% of melanoma patients experience pseudoprogression after starting ICIs treatment. Pseudoprogression is discovered with no tumor progression and is often associated with better long-term survival (15). The increased tumor volume shown by imaging examination probably owes to the recruitment of activated T cells at the tumor site during ICIs treatment. Before these cells exert their anti-tumor functions, they lead to inflammation and developed tumor volume as well as immune infiltration, edema, and necrosis. The incidence of pseudoprogression varies among tumor types but is rarely >10% (16–19). Pseudoprogression often occurs after initial ICIs treatment. It is not specific to ICIs but is more common in ICIs treatment (17).

Pseudoprogression, as an unusual but beneficial response pattern of ICIs treatment, should be emphasized and carefully recognized in clinical trials. To assist oncologists screen out patients more likely to experience pseudoprogression rather than real progression, auxiliary examinations including radiological evidence, biomarker predictors and biopsies are very useful. Only with correct diagnosis can we avoid incorrect discontinuation of effective ICIs treatment (20). Patients diagnosed as pseudoprogression can be considered for continuation of ICIs when matching all the exhibiting criteria: no severe irAEs, well-tolerated, improvement of tumor-related symptoms and imaging progression but stable condition, etc.

### Hyperprogression

Some patients can be discovered with accelerated disease progression after the initiation of ICIs therapy, thus the concept of hyperprogression was proposed (21). There is no standardized definition of hyperprogression, and the definitions varies in different studies. In the research of Champiat et al. (22), hyperprogression was defined as a Response Evaluation Criteria in Solid Tumors (RECIST) progression at the first evaluation and at least a two-fold increase of the tumor growth rate (TGR) upon prior anti-PD-1/PD-L1 therapy. A retrospective study indicated that patients who developed hyperprogression upon ICIs treatment within six weeks had worse median OS compared to patients with typical progression (23).

In the perspective of hyperprogression, both the patient’s survival and access to other alternative treatments are limited. A case report revealed that a lung cancer patient’s rib metastasis progressed rapidly after receiving ICIs-based combination therapy, and the diagnosis of hyperprogression was then set with early imaging and pathological examinations. Significant shrinkage of the metastatic lesion occurred after one month timely salvage treatment (24). For patients receiving ICIs-based combination therapy, it is necessary to make a rigorous follow-up regimen. To achieve symptom relief and longer OS in cancer patients, early detection and intervention of hyperprogression are crucial. More researches are indispensable to explore the molecular and immunological mechanisms of hyperprogression, favoring predicting and avoiding hyperprogression induced by ICIs treatment (17).

Given the perspective of clinical practice, it is necessary to figure out whether a rapid progression is hyperprogression or not. Once progression occurs, patients should be reassessed immediately and prepared to transfer to the salvage therapeutic strategy. When hyperprogression is confirmed, ICIs treatment should be stopped as soon as possible, followed by a radiologic examination to assess the patient’s condition and decide the treatment alternatives. Chemotherapy could allow a rapid tumoral response before the timepoint of the anti-tumor immune response, or even counterbalance the deleterious effect of ICIs treatment (25). As a result, combining ICIs with chemotherapy may be a helpful strategy for preventing and reversing hyperprogression.

### Dissociated response

The dissociated response is characterized by some portion of tumor lesions progressed while the other portion shrank after ICIs treatment. This kind of response pattern is similar to mixed responses seen with chemotherapy and targeted therapy (26). The dissociated response is mainly due to the tumor cells in the individual undergoing multiple divisions and proliferation during the growth process, leading to molecular biological or genetic changes in daughter cells, which consequently contributes to variances in drug sensitivity (27). The standard definition of a dissociated response needs to be further clarified. According to the RECIST version 1.1, a dissociated response is defined as an increase of some tumor lesions >20% and a shrinkage of other tumor lesions >30% (28).

The dissociated response is discovered a relatively common and unique response pattern during ICIs treatment. It is regarded as a preferable treatment response and a signal of better clinical prognosis which brings longer OS than typical progression (29). When dissociated response occurs during ICIs treatment, continuous ICIs can often evolve into a durable response (30). A specific classification for tumor lesions with the dissociated response is necessary to guide the ICIs treatment (28): As for patients with a dissociated response (1), When the clinical condition remains stable and the number of progressive lesions is limited, maintenance ICIs is recommended (2); When a minority of metastatic lesions continues to progress on CT or PET/CT, suggesting persistent immunotherapy resistance, but the rest of the metastatic lesions are in remission, local treatment in conjunction with systemic ICIs treatment can be considered (3); When different metastatic lesions activate immune in rotation (similar to a pseudoprogression pattern), ICIs are recommended maintained without local treatment.

## IrAEs determine the duration of ICIs

While achieving good efficacy, ICIs treatment may lead to some irAEs. The longer patients are on ICIs treatment, the more likely they are to develop irAEs. In most cases, irAEs emerge within 1-6 months after the initiation of ICIs treatment. Favara et al. (31) put forward that 91 days is the median onset time of irAEs at any grade. In a retrospective study, 75.8% of patients with advanced melanoma treated with ICIs experienced irAEs of any grade. The majority of irAEs appeared during the first treatment cycle, but only a small percentage (11.2%) occurred after ICIs treatment. Mild grade 1-2 irAEs tended to appear within the first two months of ICIs treatment, while grade 3-4 irAEs appeared later. There is no significant correlation between ICIs duration and irAEs severity (32). Late-onset irAEs are irAEs that occur after ICIs have been stopped (33). Previous oncological drug administration before ICIs treatment is a significant risk factor for late-onset irAEs over two years after beginning ICIs treatment (34). Therefore, it is reasonable to discontinue ICIs to avoid irAEs after achieving CR.

IrAEs often result in the discontinuation of ICIs treatment and the administration of immunosuppressant therapies. The best strategy to manage irAEs is to identify them early and stop ICIs as soon as possible, which helps to avoid or minimize the risk of rare fatal outcomes (33). The 2021 Chinese Society of Clinical Oncology (CSCO) immune checkpoint inhibitor-related toxicity management guideline (35) clearly states that when different doses and dosage forms of glucocorticoids and other immunosuppressive agents are properly combined, irAEs can usually be well managed. However, long-term use of drugs such as glucocorticoids has a risk of toxicity and may be associated with poorer survival outcomes.

Management of irAEs and ICIs treatment are not completely contradictory. When G1 irAEs appear, ICIs treatment can usually be continued while treating the toxic side effects. When G2 irAEs appear, ICIs treatment generally needs to be suspended while managing toxic side effects. In addition to certain cases, when the G2 irAEs reduce to ≤ G1, the resumption of ICIs is worth considering. After G3-G4 irAEs are properly treated, especially for G3-G4 cardiotoxicity, pulmonary toxicity, and neurotoxicity, it is generally recommended that ICIs should never be restarted. According to retrospective research, 68 (14%) of NSCLC patients treated with anti-PD-L1 therapy discontinued due to irAEs and 38 (56%) of these patients restarted ICIs after treating irAEs (36). Since the optimal duration of ICIs is unknown, the retreatment of ICIs following irAEs remission remains controversial.

## Tumor stage determines the duration of ICIs

Current clinical trials show that the duration of ICIs varies depending on the tumor stage. A brief summary is as follows (37) (**Table 1**).

**Table 1:** Duration of ICIs for different tumors and stages.

Tumor stages	Treatment
Advanced NSCLC	Two years ICIs treatment
Advanced hepatocellular carcinoma and renal carcinoma	Two-year ICIs in combination with anti-angiogenic therapy
Advanced pleural mesothelioma, malignant melanoma, and colorectal cancer	Two years of dual immunotherapy
Locally advanced tumors	Two years of consolidation immunotherapy after concurrent chemoradiotherapy or sequential chemoradiotherapy
Early and middle stage tumors	Preoperative neoadjuvant therapy: 2-4 cycles of ICIs combined with chemotherapy followed by surgery, as well as one year of adjuvant ICIs after surgery
Early and middle stage tumors	Post-operative adjuvant therapy: one year of ICIs treatment

NSCLC, non-small cell lung cancer; ICIs, immune checkpoint inhibitors.

First or second-line treatment for patients with advanced tumors. Most clinical studies in advanced tumors are currently set up for two years of ICIs treatment. Taking advanced NSCLC as an example, based on available clinical studies, it is recommended to use ICIs for two years among first-line monotherapy, second-line monotherapy, first-line immunotherapy combined with chemotherapy, and dual immunotherapy (38). The National Comprehensive Cancer Network (NCCN) guidelines recommend that patients with NSCLC should receive maintenance ICIs therapy for 2 years if they received first-line immunotherapy (39). For advanced liver cancer and renal cancer, a two-year combination of ICIs and anti-angiogenic therapy is the main first-line treatment option. In addition, dual immunotherapy has been approved as a first-line treatment for various cancers, including advanced renal cancer, NSCLC, pleural mesothelioma, malignant melanoma and colorectal cancer, with the same recommendation of two years duration. After two years of ICIs treatment, drug withdrawal can be considered; if the patient desires to continue ICIs treatment, consent can be provided in principle.

Consolidation immunotherapy for patients with locally advanced tumors. The duration is usually 1-2 years. The PACIFIC study aims to evaluate the efficacy of consolidation therapy with durvalumab in patients with locally advanced NSCLC who have not experienced disease progression after concurrent chemoradiotherapy with platinum-containing regimens. In 2017, the study published the first results that PFS was significantly longer in the one-year group on durvalumab consolidation after concurrent chemoradiotherapy than in the placebo group, which quickly changed the clinical practice. Recently, the study reported a 42.9% five-year survival rate, with 1/3 of patients still alive after five years of PFS (40). However, there is no sufficient evidence of other tumor types. The newly published GEMSTONE-301 study recommended two years of consolidation immunotherapy after concurrent chemoradiotherapy or sequential chemoradiotherapy (41). For NSCLC, the existing guidelines recommend 2 years of ICIs therapy, with an overall fair safety profile and infrequent occurrence of irAEs. Therefore, a 2-year duration of consolidation immunotherapy is strongly recommended.

Preoperative neoadjuvant therapy for patients with early and middle stage tumors. In recent years, neoadjuvant therapy with ICIs alone or in combination with chemotherapy or dual immunotherapy has been used to treat tumors like NSCLC, triple-negative breast cancer, and esophageal cancer in several clinical studies. The major pathological remission (MPR) of patients who underwent surgery was twice that of neoadjuvant chemotherapy, and the safety was good. In 2021, a Phase III clinical trial CheckMate 816 reported that nivolumab combined with chemotherapy neoadjuvant therapy showed a significant improvement in pathological CR rates (42). The FDA approved nivolumab in combination with platinum-containing dual chemotherapy for the neoadjuvant treatment of adult patients with resectable NSCLC on March 4, 2022, based on the Phase II clinical trial NADIM. Surgery is currently recommended after 2-4 cycles of ICIs combined with chemotherapy for NSCLC, triple-negative breast cancer and esophageal cancer. One year of adjuvant ICIs treatment is recommended following surgery. In addition, there are also other alternative options.

Postoperative adjuvant therapy for patients with early and middle stage tumors. The duration is usually one year. The therapy is applied in various tumors such as esophageal cancer, breast cancer, malignant melanoma, uroepithelial cancer, renal cancer, etc. Taking NSCLC as an example, based on the IMpower 010 study, on March 16, 2022, the National Medical Products Administration (NMPA) approved atezolizumab as adjuvant therapy for patients with stage II-IIIA NSCLC with ≥ 1% tumor cell PD-L1 expression, surgically removed and platinum-based chemotherapy (43). Recently, the KEYNOTE-091 study also demonstrated that pembrolizumab in combination with or without adjuvant chemotherapy significantly improved disease-free survival in patients with stage IB-IIIA NSCLC after surgical resection, regardless of PD-L1 expression level.

## Debate for limited or continuous ICIs

In the medical community, there exists a pair of opposite perspectives on the optimal duration of ICIs. On the one hand, ICIs treatment induces a durable response in the body, allowing the previously activated immune system to regress tumor growth. In addition, short-term ICIs treatment can also avoid the toxic side effects attributed to long-term use. Therefore, some experts advocated discontinuing ICIs after a period of treatment. On the other hand, insufficient ICIs treatment duration may result in disease progression or relapse following remission. Therefore, other experts advocated continuing ICIs treatment to improve patients’ long-term PFS and OS. Numerous clinical trials and other studies have set the duration of ICIs, thus we can determine the optimal duration of ICIs more properly based on the results of these trials. The results of these clinical trials are shown in **Table 2**. During the European Lung Cancer Congress 2022, a session was allocated to this topic for debate and voting by the conference committee.

**Table 2:** Clinical trials investigating the duration of ICIs.

Trials	Cancer	Phase/Size	ICIs	Duration	Results
The Safe Stop trial (NL7293) (3)	Melanoma	N=200	Anti-PD-1	1 year Until CR or PR	NR NR
CheckMate153 (NCT02066636) (11)	NSCLC	III (N=1434)	Nivolumab	Until progression, unacceptable toxicity, or withdrawal of informed consent 1-year-fixed duration	PFS: 24.7m OS: NR PFS: 9.4m OS: 32.5m
CheckMate067 (NCT01844505) (12)	Melanoma	III (N=1296)	Nivolumab and Ipilimumab Nivolumab Ipilimumab	Until progression, unacceptable toxicity, or withdrawal of informed consent	OS: NR 3-year OS: 58% OS: 37.6m 3-year OS: 52% OS:19.9m 3-year OS: 34%
KEYNOTE-024 (NCT02142738) (38)	NSCLC	III (N=305)	Pembrolizumab	2 years	PFS:10.3m OS:26.3m 5-year OS: 31.9%
KEYNOTE-042 (NCT03850444) (38)	NSCLC	III (N=262)	Pembrolizumab	2 years	PFS: 5.4m OS: 16.7m
KEYNOTE-189 (NCT03950674) (38)	NSCLC	III (N=40)	Pembrolizumab	2 years	PFS: 9.0m OS: 22m ORR: 85.7%
KEYNOTE-407 (NCT03875092) (38)	NSCLC	III (N=125)	Pembrolizumab	2 years	PFS: 6.4m OS: 15.9m
IMpower110 (NCT02409342) (38)	NSCLC	III (N=572)	Atezolizumab	Until progression, unacceptable toxicity, or death (maximum up to approximately 58 months)	PFS: 5.7m OS: 20.2m
IMpower130 (NCT02367781) (38)	NSCLC	III (N=723)	Atezolizumab	Until progression	PFS: 7.0m OS: 18.6m
IMpower150 (NCT02366143) (38)	NSCLC	III (N=1202)	Atezolizumab	Until progression	PFS: 8.3m OS: 19.8m
CheckMate227 (NCT02477826) (38)	NSCLC	III (N=2748)	Nivolumab and Ipilimumab	Until progression, unacceptable toxicity, or for 2 years	PFS: 5.1m OS: 17.1m
CheckMate9LA (NCT03215706) (38)	NSCLC	III (N=719)	Nivolumab and Ipilimumab	Until progression, unacceptable toxicity, or for 2 years	OS: 15.6m
PACIFIC (NCT04230408) (40)	NSCLC	III (N=48)	Durvalumab	1 year	PFS: 16.9m OS: 47.5m
GEMSTONE-301 (41)	NSCLC	III (N=381)	Sugemalimab	2 years	PFS: 9.0m
CheckMate816 (NCT02998528) (42)	NSCLC	III (N=505)	Neoadjuvant Nivolumab	Until surgery	EFS: 31.6m
IMpower010 (NCT02486718) (43)	NSCLC	III (N=1280)	Atezolizumab	1 year	HR for DFS: 0.81 (0·67-0·99; p=0·040)
NCT0267397 (44)	Melanoma	N=200	Pembrolizumab or Nivolumab	1 year	ORR: 96%
The DANTE trial (ISRCTN15837212) (45)	Melanoma	III (N=1208)	Anti-PD-1	Until progression, unacceptable toxicity, or for 2 years	NR
KEYNOTE-001 (NCT01295827) (46)	NSCLC	I (N=550)	Pembrolizumab	Until progression, unacceptable toxicity, or for 2 years	OS: 22.3m 5-year OS: 29.6%
CA209-003 (NCT00730639) (47)	NSCLC	I (N=395)	Nivolumab	Until progression, unacceptable toxicity, confirmed CR, or for 2 years	5-year OS: 16%
Mäkelä et al. (48)	Melanoma	N=40	Anti-PD-1	6 months	PFS: 12m OS: NR
KEYNOTE-006 (NCT01866319) (49)	Melanoma	III (N=834)	Pembrolizumab Ipilimumab	2 years	PFS: 8.4m OS: 32.7m PFS: 3.4m OS: 15.9m
KEYNOTE-010 (NCT01905657) (50)	NSCLC	II/III (N=1034)	Pembrolizumab	2 years	3-year OS: 83.0% 5-year OS: 25.0%
NCT01693562 (51)	Various	I/II (N=1022)	Durvalumab	Retreatment after 1 year	PFS: 5.9m OS: 23.8m

ICIs, immune checkpoint inhibitors; m, months; NSCLC, non-small cell lung cancer; NR, not reached; N, number; PFS, progression-free survival; OS, overall survival; ORR, overall response rate; EFS, event-free survival; DFS, disease-free survival; HR, hazard ratio.

### Limited ICIs treatment

At present, more studies are standing for this view. Jansen et al. (44) found that in 185 patients with advanced melanoma who had accepted one year of pembrolizumab treatment, the risk of disease recurrence was low when treatment is stopped after achieving CR, and the risk of progression was reduced in patients who had CR for more than six months. But patients who achieved PR or SD were more likely to relapse after discontinuation. Patients who discontinued pembrolizumab after achieving SD had a high risk of disease progression, thus effective ICIs treatment should not be discontinued unless there occurred fatal irAEs (45). Similarly in NSCLC, a real-world study noted that duration of disease control after ICIs discontinuation was correlated with tumor response situation when treatment discontinued, and these results called for caution in discontinuing treatment in patients with SD as the best response (46). In the KEYNOTE-001 study, patients who stopped taking pembrolizumab after achieving CR had an 89.9% disease-free survival rate after 24 months (9). A real-world study showed that patients who responded early to ICIs had a longer OS and a lower risk of disease progression when they discontinued ICIs after achieving CR (47). A multicenter retrospective study (KCSG LU20-11) reported the long-term follow-up results in patients with advanced and/or metastatic NSCLC. It was found that a significantly high proportion of patients who completed 2 years of ICIs therapy continued to experience long-term PFS. Even if ICIs were discontinued in patients without disease progression after 6 months administration, they might achieve a durable response and facilitate long-term survival (48). In an observational cohort study, 52 patients with metastatic melanoma who discontinued anti-PD-1 therapy after one year remained free of disease progression in the long-term follow-up, and the risk of disease progression was low even in patients with remnant lesions by imaging (49). It has been shown that when the active disease is not detected on CT or PET/CT scans or biopsies, discontinuing anti-PD-1 therapy after 12 months may result in a lower rate of disease recurrence in patients with advanced melanoma (50). In a retrospective study by Valentin et al., patients with advanced melanoma who discontinued anti-PD-1 therapy for reasons other than disease progression were shown to have durable responses with a disease recurrence rate of only 18.5% (51). A real-world multicentric observational study including 1011 patients in India showed that short-course ICIs therapy had comparable efficacy/safety to standard ICIs therapy (52). Oulu University Hospital retrospectively collected all patients who had been treated with anti-PD-1 therapy for metastatic disease in lung and genitourinary (renal and bladder) cancers as well as melanoma, with maximal anti-PD-1 therapy length restricted to 6 months, turning out 11 of 17 responders who discontinued anti-PD-1 therapy after 6 months therapy remained SD after 1 year (53). The above studies all suggest that discontinuation of anti-PD-1 therapy may be attempted in specific populations.

To verify the above, there are at least two prospective investigations currently in progress. The DANTE trial was designed to determine whether time-limited therapy could improve clinical outcomes by reducing toxicities while maintaining treatment benefits. The results supported time-limited therapy for patients with metastatic melanoma continuously remaining progression-free after two years of ICIs (54). The Dutch Safe Stop trial will confirm the feasibility of early discontinuation of ICIs by assessing sustaining response rates after discontinuing first-line nivolumab or pembrolizumab monotherapy in patients with advanced melanoma who achieve CR or PR (3).

Therefore, there is a premature suggestion for melanoma-early discontinuation of ICIs can be considered in CR patients ready to receive additional treatment for 6 months after achieving CR (55). However, unlike melanoma, CR as a sign for treatment cessation has not been widely adopted in advanced NSCLC due to the low CR rates (< 5%) (56). In the CA209-003 study, more than 75% of NSCLC patients treated with 96 weeks of time-limited nivolumab showed a five-year PFS (57). For patients with advanced NSCLC, a treatment regimen of up to two years of ICIs is still widely recommended.

### Continuous ICIs treatment

Despite the perspective of limited ICIs treatment, other studies have suggested that stopping ICIs after two years of treatment may result in disease progression. In the KEYNOTE-189 study, half of the 56 patients who completed 35 cycles (approximately two years) of pembrolizumab progressed after stopping ICIs treatment (58). In the KEYNOTE-010 study, 25 patients (32%) experienced disease progression after stopping treatment with 35 cycles of pembrolizumab (59). Similarly, 54% of 39 patients treated with 35 cycles of pembrolizumab had disease progression two years after stopping ICIs treatment (60).

Arbitrary discontinuation may result in disease relapse in the absence of reliable response markers and predictors of long-term benefit. In a prospective trial, 17 patients dropped out after six months of anti-PD-1 treatment, and 14 (82%) of those experienced relapse (61). In the phase I clinical trial, patients with solid tumors who were treated with ICIs for < 12 months had a higher rate of disease recurrence than those who were treated for > 12 months, and disease recurrence often occurred during the early post-treatment discontinuation period (62). According to a study on advanced melanoma, patients with advanced tumors and those whose best response is not CR should receive ICIs for a longer duration and should not discontinue ICIs before 18 months (63).

So, is two years of ICIs really the best option for patients with advanced tumors? Data from patients in the CheckMate 153 trial, in which patients with NSCLC responding to anti-PD-1 therapy were randomly assigned to one year versus continuous nivolumab, suggested that the median PFS and OS were longer for continuous ICIs treatment group (11). This study also supported the administration of nivolumab for more than one year in previously treated patients with advanced NSCLC. According to a long-term analysis of KEYNOTE-010, 91.0% (72/79) of the 79 patients who completed two years of pembrolizumab therapy survived, with an estimated 24-month OS rate of 86.3% (59). In existing clinical protocols, anti-PD-1 monoclonal antibodies are generally administered for two years or longer (64). A study showed that continuous ICIs treatment for more than two years resulted in higher 3-year OS rates (85.7% *vs*. 100%, 2-year group *vs*. > 2-year group) and the lower 3-year OS rate in the < 2-year group (49%), suggesting that the clinical benefit is likely to be seen in patients who had been on continuous treatment for more than two years (65). However, longer ICIs treatment also contributes to more severe irAEs. Clinical practitioners must weigh the benefits of therapy duration against the risks of toxicities.

**Figure 2:**
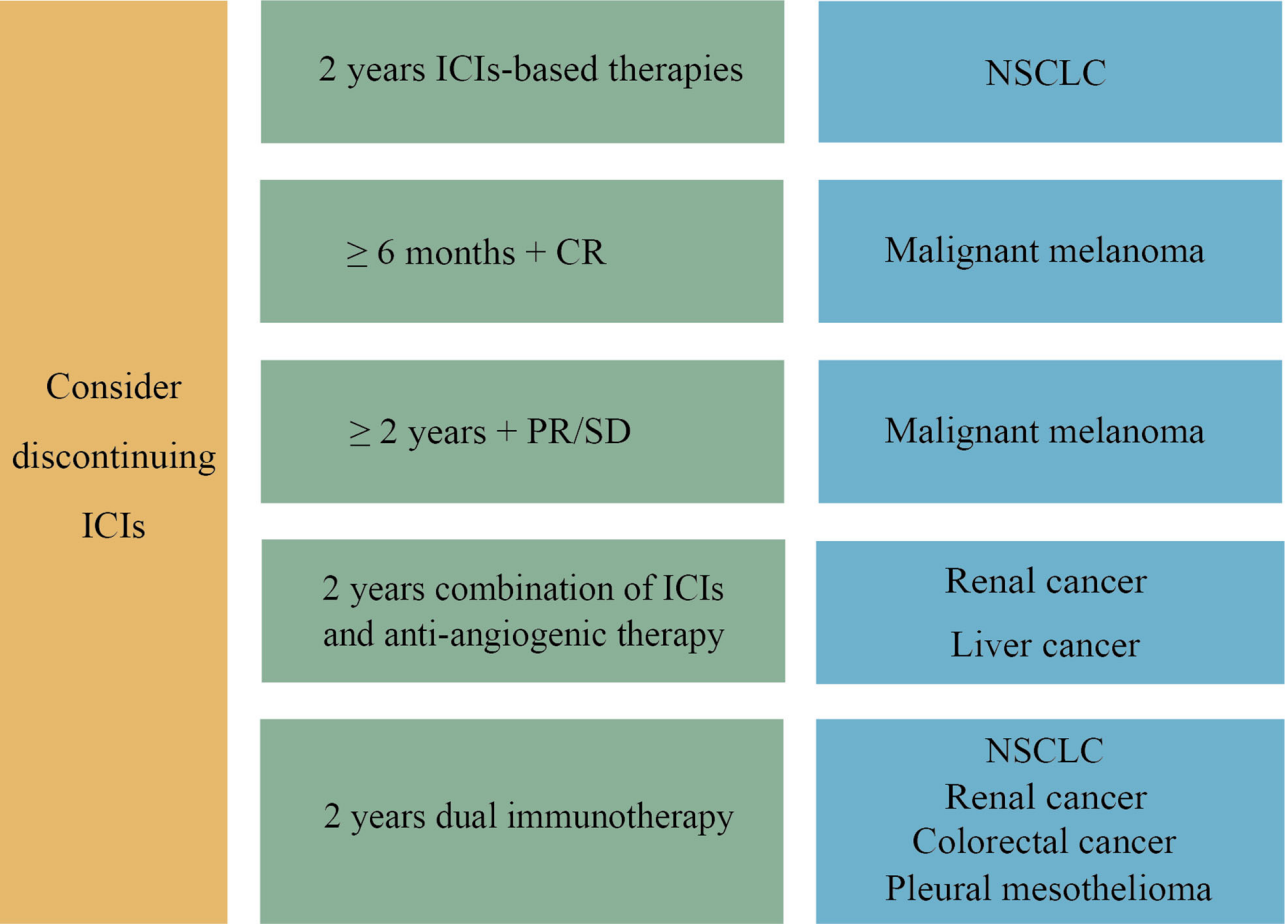
The optimal duration of ICIs in different tumor types. For NSCLC, we recommend that discontinuation be considered when 2 years of ICIs-based therapy are completed. For advanced malignant melanoma, early discontinuation of ICIs can be considered in CR patients ready to receive additional treatment for 6 months after achieving CR. If the efficacy is assessed as PR or SD after two years of ICIs treatment, cessation may be taken into account. A two-year combination of ICIs and anti-angiogenic therapy is the main first-line treatment option for advanced liver cancer and renal cancer. In addition, dual immunotherapy has been approved as a first-line treatment for various cancers, including NSCLC, advanced renal cancer, colorectal cancer and pleural mesothelioma, with the same recommendation of two years duration. After two years of ICIs treatment, drug withdrawal can be considered.

Dual immunotherapy causes more severe irAEs than immune monotherapy or immunotherapy combined with chemotherapy. Hence, the optimal duration of dual immunotherapy also needs to be clarified. Prospective studies are currently being conducted to determine the appropriate duration of combination immunotherapy. In the phase III DISCIPLE (NCT03469960) study to determine the optimal duration of dual immunotherapy of ipilimumab and nivolumab in patients with advanced NSCLC, patients who do not progress after six months of dual immunotherapy will be randomly assigned to a group to continue ICIs treatment until disease progression, or to the other group to stop ICIs treatment (1). A figure displaying the optimal duration of ICIs based on tumor types was composed for consultation (**Figure 2**).

### ICIs re-challenge

The problems associated with long-term continuous ICIs treatment include the potential risk of late toxicity, the financial burden of the high cost and the poor life quality of patients due to irAEs, etc. There emerge growing interests in two aspects: predicting the long-term prognosis of discontinuing ICIs, and re-challenging anti-PD-1 therapy when the disease progresses.

Restarting ICIs when disease progresses in patients who initially benefited from ICIs treatment is considered safe and effective and can achieve disease control during ICIs treatment. In the phase III KEYNOTE-006 study, 12 of 27 patients who progressed after completing two years of pembrolizumab treatment were re-treated with pembrolizumab, and the best overall response was 3 CR, 3 PR, 3 SD, 1 PD, and 2 with evaluation pending (66). In the KEYNOTE-010 trial, 21 patients who progressed after completing two years of pembrolizumab restarted ICIs treatment, reporting that 11 (52.4%) had objective responses and 15 (71.4%) were alive at the time of data cutoff (67). In a trial of patients treated with durvalumab for one year and then discontinued, 71 patients experienced disease progression during that time and restarted durvalumab treatment, with more than 70% of patients experiencing clinical benefit (68). In the study by Warner et al., 15% of patients responded to re-treatment with anti-PD-1 therapy and 25% responded to re-treatment with the combination of ipilimumab and nivolumab (69). In addition, patients who have suspended ICIs because of irAEs need to be aware of the following four points before restarting ICIs treatment (70–72) (1): Population selection. If patients have responded to ICIs (CR or PR) before the appearance of irAEs, there is no need to restart once the irAEs have been resolved. Conversely, ICIs treatment should be restarted if there is no tumor response. It is conceivable that patients who develop irAEs while receiving ICIs treatment have a high immune response (2). Informed consent. Restarting ICIs treatment increases the likelihood of irAE recurrence by roughly 50%. Recurring irAEs can manifest as either familiar or unexpected symptoms. If hospitalization is required when irAEs occur for the first time, irAEs are more likely to occur when ICIs are used again. As a result, obtaining informed consent from the patient is critical before cautiously beginning. If irAEs recur after a restart, the treatment protocol is the same as before, but this type of ICIs should be stopped permanently (3). Treatment principles for restarting ICIs varies when previous irAEs organs are diverse. Taking into account irAEs in different organs, restarting ICIs requires distinct considerations, including the indication for restart. Therefore, a specialist consultation should be invited before restarting ICIs treatment. For further information, see the 2021 CSCO immune checkpoint inhibitor-related toxicity management guideline (35) (4). When restarting, try to choose ICIs distinct from previous treatment. For example, if a patient has developed grade 3 or 4 toxicity with an ipilimumab-containing regimen, further treatment may include PD-1 or PD-L1 inhibitor monotherapy after the early toxicity is eliminated.

## Conclusion and perspectives

According to existing studies, there is no conclusive evidence regarding the optimal duration of ICIs. A growing number of studies have explored the timing of discontinuing and restarting ICIs in malignant melanoma and NSCLC based on efficacy and irAEs, but there is not yet sufficient evidence to answer this question. For melanoma, the recommended optimal duration of ICIs is an additional 6 months of ICIs treatment after the patient achieving CR. The existing consensus suggests that the optimal duration of ICIs should be considered based on the response pattern, irAEs, and tumor stages. Meanwhile, combining some necessary examinations such as PET-CT, liquid biopsy (e.g., ctDNA) or tissue biopsy can help determine when to discontinue ICIs. As more and more prospective studies are completed and published, the optimal duration of ICIs will be found.

## Author contributions

JY, YS and JT collected and analyzed the literatures. JY and YS drafted the manuscript. JT revised the manuscript. BZ came up with the origin idea and supervised the work. All authors contributed to the article and approved the final version.

## Funding

This research was funded by National Natural Science Foundation of China (Grant No. 82272928), CSCO-BMS Cancer Immunotherapy Research Foundation (Grant No. Y-BMS2019-003) and Wuhan Municipal Science and Technology Bureau Knowledge Innovation Special Project (Grant No. 2022020801010475).

## Conflict of interest

The authors declare that the research was conducted in the absence of any commercial or financial relationships that could be construed as a potential conflict of interest.

## Publisher’s note

All claims expressed in this article are solely those of the authors and do not necessarily represent those of their affiliated organizations, or those of the publisher, the editors and the reviewers. Any product that may be evaluated in this article, or claim that may be made by its manufacturer, is not guaranteed or endorsed by the publisher.
